# A One-Degree-of-Freedom Test for Supra-Multiplicativity of SNP Effects

**DOI:** 10.1371/journal.pone.0078038

**Published:** 2013-10-30

**Authors:** Christine Herold, Alfredo Ramirez, Dmitriy Drichel, André Lacour, Tatsiana Vaitsiakhovich, Markus M. Nöthen, Frank Jessen, Wolfgang Maier, Tim Becker

**Affiliations:** 1 German Center for Neurodegenerative Diseases (DZNE), Bonn, Germany; 2 Department of Biostatistics, Harvard School of Public Health, Boston, Massachusetts, United States of America; 3 Institute for Medical Biometry, Informatics and Epidemiology, University of Bonn, Bonn, Germany; 4 Department of Psychiatry and Psychotherapy, University of Bonn, Bonn, Germany; 5 Institute of Human Genetics, University of Bonn, Bonn, Germany; 6 Department of Genomics, Life and Brain Center, University of Bonn, Bonn, Germany; INRCA, Italy

## Abstract

Deviation from multiplicativity of genetic risk factors is biologically plausible and might explain why Genome-wide association studies (GWAS) so far could unravel only a portion of disease heritability. Still, evidence for SNP-SNP epistasis has rarely been reported, suggesting that 2-SNP models are overly simplistic. In this context, it was recently proposed that the genetic architecture of complex diseases could follow limiting pathway models. These models are defined by a critical risk allele load and imply multiple high-dimensional interactions. Here, we present a computationally efficient one-degree-of-freedom “supra-multiplicativity-test” (SMT) for SNP sets of size 2 to 500 that is designed to detect risk alleles whose joint effect is fortified when they occur together in the same individual. Via a simulation study we show that the SMT is powerful in the presence of threshold models, even when only about 30–45% of the model SNPs are available. In addition, we demonstrate that the SMT outperforms standard interaction analysis under recessive models involving just a few SNPs. We apply our test to 10 consensus Alzheimer’s disease (AD) susceptibility SNPs that were previously identified by GWAS and obtain evidence for supra-multiplicativity (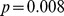) that is not attributable to either two-way or three-way interaction.

## Introduction

Despite of thousands of confirmed disease susceptibility variants [1], the findings from Genome-wide association studies (GWAS) so far explain only a portion of the heritability of complex diseases [2]. Multi-SNP approaches like interaction and pathway analysis were proposed [3] to detect the still unexplained portion of genetic disease risk. While Genome-wide interaction analysis has become computationally feasible [4], [5], by now only few, if any, replicable interactions have been found. In order to explain the phenomenon of missing evidence for interaction, Zuk et al. [6] suggested that common diseases may follow so-called limiting pathway liability models (LPLMs). A LPLM is defined by multiple risk factors which imply a risk threshold. Individuals with a risk allele load above the threshold have a strongly increased disease risk, while a baseline risk applies below the threshold. LPLMs can be viewed as a special case of the larger class of liability models [7], [8] which allow that the risk contribution of the involved factors may vary. In addition, the LPLMs focus on a single pathway that is under polygenic influence. In contrast to that, Li et al. [8] describe two sources of liability to depression, namely genetic liability for stress sensitivity mediating depression, and genetic liability for depression in general. Both sources are shown to be under polygenic control. A key feature of these models and the simpler LPLMs is that they imply epistasis that goes beyond two-way interaction. Further important classes of more complex high-dimensional models have been discussed in [9].

Although pointed out previously [10], it is worthwhile to recall that diverging definitions and interpretation of the terms “interaction” or “epistasis” in the literature often lead to confusion. The topic is intrinsically difficult, since the statistical definition of interaction is scale-dependent [11]. In this paper, as in the majority of statistical publications on the topic, we interpret interaction as deviation from multiplicative relative risks, which corresponds to deviation from additivity on the logarithmic scale used in logistic regression models. This definition is the appropriate definition for rare diseases [12] and will also prove to be appropriate in the settings we are going to investigate.

The risk allele threshold models proposed by Zuk et al. [6] lead to marginal effects that are comparable with effect sizes observed in GWAS studies and imply both low and high-dimensional interactions. However, pairwise interaction, although present, is typically so small that it would be detectable only with sample of several hundreds of thousands of individuals. In this sense, LPLMs would be consistent with the expected importance of genetic interaction [10], [13], [14] on the hand and lacking statistical evidence for its presence on the other hand.

The search for deviation from multiplicativity in all medium-sized SNP sub sets of a GWAS panel is clearly unfeasible and not a realistic strategy in the coming years. However, it is an important research question how to decide whether a *given* set of SNPs displays “supra-multiplicativity” of allelic risks. In this paper, we present a powerful one degree of freedom (d.f.) regression test for deviation from multiplicativity which simultaneously addresses interactions of all orders and which is particularly powerful in the presence of threshold models.

## Results

### Empirical Levels

Table 1 shows results from the simulations under the model with marginal effects, but no interaction effects of any kind. Under all scenarios, the empirical levels are slightly lower than the nominal level. This phenomenon is caused by the application of a Bonferroni-correction to not completely independent test statistics. The conservativeness is significant for 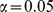 and all SNP sizes, as well as for 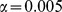 and for SNP sets with less than 30 SNPs. However, the observed conservativeness is rather small in size. The strongest difference we observe is an empirical level of 0.042 at 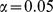 for 40 SNPs. Therefore, we conclude that the application of the Bonferroni-correction is sufficient for practical purposes. The results of the power will support this claim.

**Table 1:** Empirical 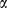-levels for supra-multiplicativity test (SMT)^a^.

	Number of SNPs				
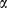 b	10	20	30	40	50
0.05	0.044*	0.045*	0.046*	0.042*	0.045*
0.005	0.0043*	0.0043*	0.0045*	0.0047	0.0049
0.0005	0.00048	0.00043	0.00043	0.00046	0.00045

a Under a model with multiplicative SNP effects, without interaction effects.

b Nominal significance level.

* indicates significant deviation from the nominal level.

In table 2, “nominal levels” or, depending on the perspective, power levels for the non-multiplicative models 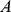 and 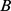 are shown. Model 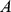 involves non-zero dominance effects and model 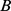 has several pairwise interactions. In other words, classic logistic regression test for dominance deviation or two-way interaction would be the method of choice. Under both models, the SMT shows a measurable excess of the nominal level, i.e., already 1-way interactions ( = dominance effects) and two-way interactions produce small signals. The level of excess of the nominal level is very small, in particular under scenario 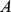. Under scenario 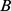 the excess is slightly more substantial, at a nominal 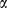 of 0.005 the empirical level is 0.021. The SMT will typically be applied to detect higher-order interactions in which case adjustment for a priori known or data-derived dominance or interactions terms is warranted. When such terms are added as additional covariate parameters, the excess of the nominal level disappears (table 2). In other words, adjustment for significant interaction terms is possible and should be applied in order to investigate which terms drive the significance of a successful SMT application.

**Table 2:** Empirical 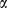-levels for SMT^a^.

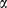 b	Model 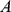	Model 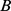
0.05	0.048/0.0047	0.123*/0.047
0.005	0.008*/0.004	0.021*/0.0045
0.0005	0.001/0.0003	0.006*/0.0004

a Under models with SNP dominance (model 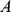) or pairwise interaction effects (model 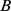), with/without dominance and interaction covariates.

b Nominal significance level.

* indicates significant deviation from the nominal level.

### Power of Single-marker Analysis and Conventional Interaction Analysis

Table 3 and table 4 show the power of conventional one-SNP-at-a-time and pairwise interaction analysis in the presence of different LPLMs. Let us first consider the penetrance value 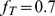. Such a penetrance might be considered to be unusually high, but leads to allele relative risks no larger than 1.4. Consistently, power for single-marker analysis in table 3 is on a level that is reasonable given a sample size of 3000 cases and 3000 controls. With 50 SNPs, there is 62% power to detect at least one SNP at the Genome-wide level of 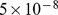. Power increases with decreasing size of the SNP set. This is not too surprising, since in our set-up we kept the portion of individuals above the liability threshold constant. As a consequence, the marginal effects become larger when fewer SNPs are part of the model. With 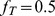, power of single-marker analysis is low for more than 30 SNPs and at 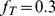, only under the 10-SNP model some power is left. However, it has to be emphasized that much higher penetrances 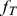 have been suggested [6] and that we included the lower 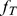 values for the purpose of completeness.

**Table 3:** Power values^a^ for single-marker analysis.

	Number of SNPs				
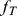 b	10	20	30	40	50
0.1	0.03	0.00	0.00	0.00	0.00
0.3	0.79	0.00	0.00	0.00	0.00
0.5	0.99	0.52	0.24	0.25	0.09
0.7	0.99	0.99	0.91	0.89	0.62

a Power to detect at least one SNP at 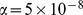.

b Penetrance for individuals above the allele load threshold.

**Table 4:** Power values^a^ for pairwise interaction.

	Number of SNPs				
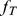 b	10	20	30	40	50
0.1	0.21	0.00	0.00	0.00	0.00
0.3	0.70	0.02	0.00	0.00	0.00
0.5	0.99	0.11	0.00	0.00	0.00
0.7	1.00	0.28	0.01	0.00	0.00

a Power to detect at least one pairwise interaction (with 1 d.f. test) at 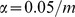, where 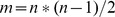 is the number of pairwise tests for 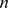 SNPs. Power at the Genome-wide significance level for interaction (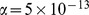) was always 0.

b Penetrance for individuals above the allele load threshold.

Power for pairwise interaction is typically absent (table 4), with the exception of the 10 SNP model and to some extent the 20 SNP model for 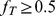. Even under the 10-SNP model interaction can be detected only at a significance level of 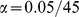, i.e., under the assumption that the 10 SNPs of the model are known a priori and that only these SNPs are tested for interaction. In a Genome-wide search it is impossible to detect any of the interactions at the experiment-wide significance level of 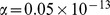. In summary, our LPLMs are consistent with the models proposed by Zuk et al. [6] and also consistent with the results of the last years of GWAS analysis: the LPLMs imply some detectable marginal effects, but pairwise interaction effects are not identifiable with the given sample size.

### Power of SMT

**Figure 1:**
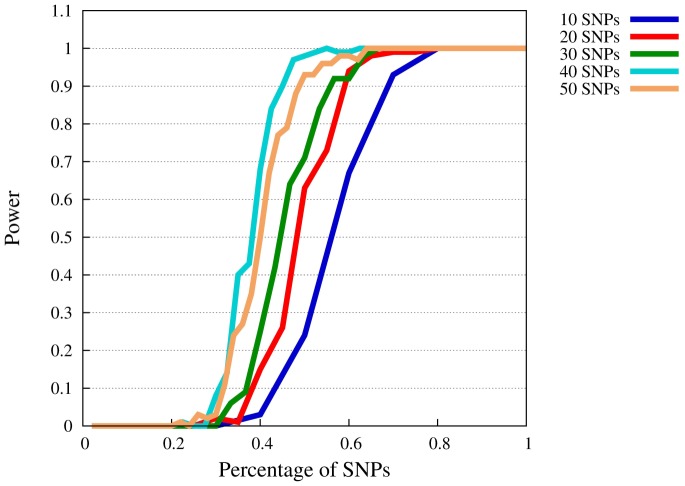
Power of SMT at 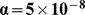 for 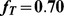. The 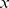-axis represents the available percentage of SNPs of the complete model, the 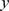-axis power levels.

**Figure 2:**
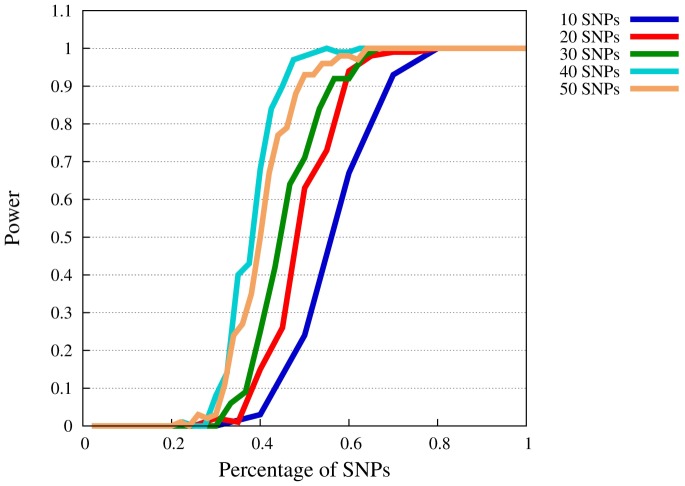
Power of SMT at 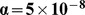 for 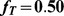. The 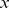-axis represents the available percentage of SNPs of the complete model, the 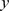-axis power levels.

**Figure 3:**
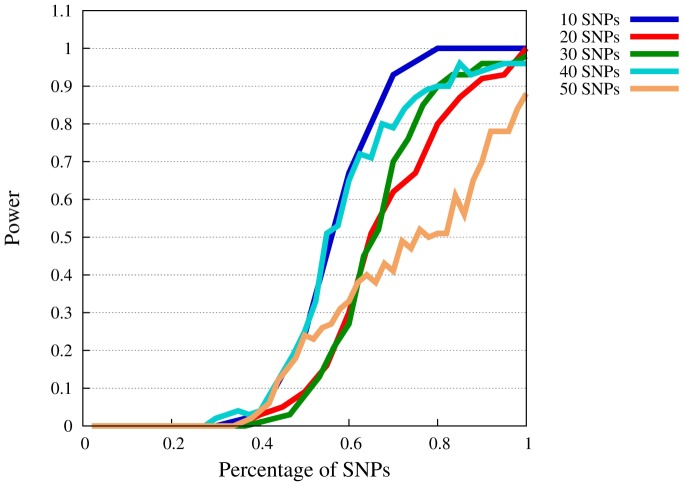
Power of SMT at 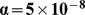 for 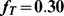. The 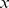-axis represents the available percentage of SNPs of the complete model, the 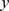-axis power levels.

**Figure 4:**
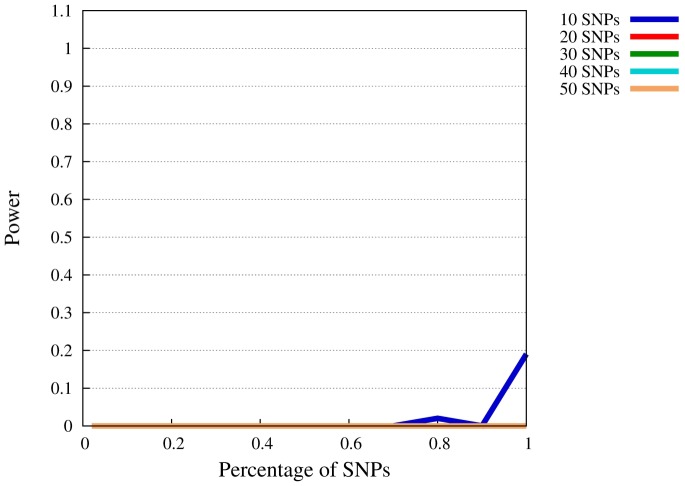
Power of SMT at 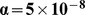 for 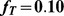. The 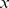-axis represents the available percentage of SNPs of the complete model, the 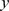-axis power levels.

Figures 1, 2, 3, and 4 show power levels of the SMT in the presence of LPLMs which follow the specifications by Zuk et al. [6]. We start with the description of the power curve for 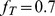 (figure 1). As expected, power increases with the percentage of model SNPs that can be included. In order to reach a power of 80%, only about 35% of the risk SNPs have to be known. Here, the required portion is smaller for the models with more SNPs, but the effects on the power curves depends only moderately on the number of SNPs in the model. The figure also shows, that below a portion of 20% of known SNPs there is typically no power to detect an involvement in a liability model.

When 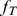 is reduced to 0.5 (figure 2), the portion of required SNPs increases as expected. However, a power level of 80% can still be reached also when only about 35% to 55% of the involved SNPs are known. In addition, all models have 100% power when more than 60% of the SNPs are included.

When 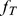 is lowered to 0.3 (figure 3), power drops markedly. As mentioned before, 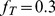 is a rather small penetrance value for a liability threshold model. We included this model for completeness and in order to show limits of our approach. For 80% power, now between 60% to 95% of the SNPs have to be known a priori. It seems unlikely that such a high portion of disease SNPs is known in advance, at least at the current state of research. For higher values of 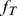, say 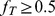, is more reasonable to assume that the required portion of SNPs is known a priori. Finally, at 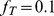 (figure 4), power vanishes completely, except for the 10-SNP model. This penetrance value, however, is so small that also single-marker analysis has no power, cf. table 3. In other words, hardly any method will allow to to detect association with the sample size investigated here.

**Figure 5:**
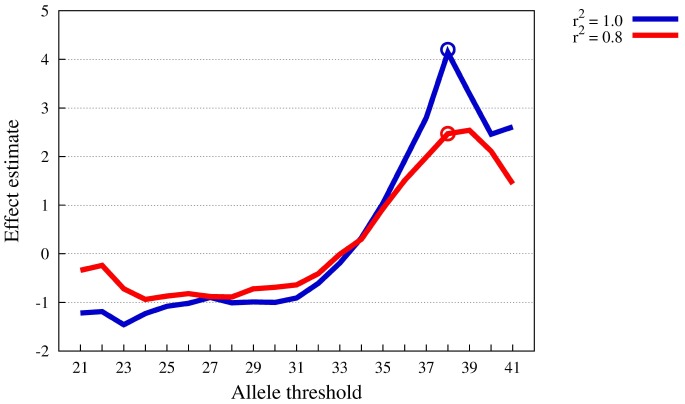
Comparison of 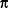-estimates between 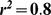 and 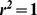 tagging of causal variants under LPLM defined by 30 SNPs, 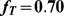. The 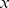-axis represents the allele threshold, the 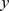-axis the supra-multiplicativity effect estimate. The circles indicate the “true” risk allele threshold.

**Figure 6:**
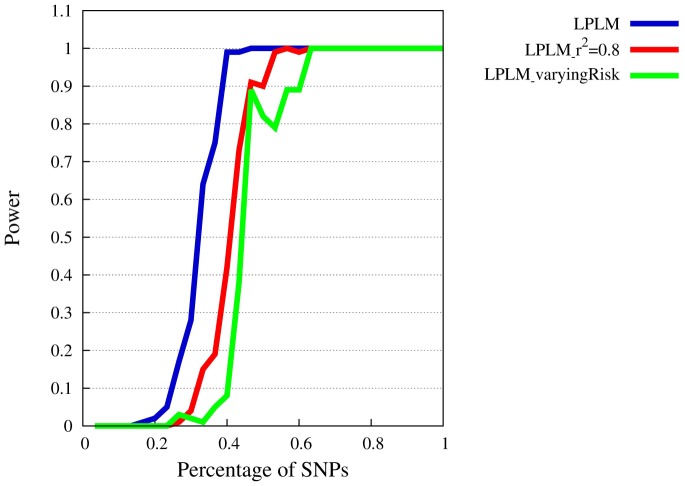
Power of SMT test at 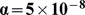 for 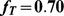. Comparison of fixed threshold and modified threshold model. The 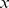-axis represents the available percentage of SNPs of the complete model, the 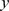-axis power levels.

In figure 5 the impact of incomplete tagging of the true causal variants (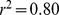 for all SNPs) is exemplified for the 30-SNP LPLM (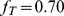). The critical allele load risk threshold under this model is 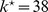. Under perfect tagging (blue curve, 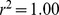), the regression estimates (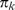) for “supra-multiplicativity” grow along with the allele load 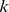 and rise to a sharp maximum at 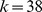 that coincides with the simulated parameter. Under imperfect tagging (red curve), the regression estimates 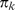 have a smaller range, which reflects the overall reduced power to detect supra-multiplicativity, c.f. also the power curve under incomplete tagging in figure 6. On the other hand, the curve displays a similar behavior as before, it rises along with 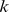 to a maximum, now at 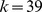. In other words, the true maximum is missed, but the peak occurs very close to the simulated parameter. We concluded that the SMT works well also when the true causal SNPs are only captured by variants in LD.

Figure 6 contrasts the power of the SMT under the 30-SNP LPLM (blue curve) to the modified LPLM, which allows variation of individual SNP risks (red curve). A slightly higher portion of known SNPs is now required to reach a given power level. The number of required SNPs increases by two on average. The SNP effects that result under the modified model are quite high (relative risks up to 2). As a consequence, a higher portion of the variance is now explained by marginal effects and the portion of variance explained by the supra-multiplicativity is reduced. In view of the fact that modified LPLM implies strong marginal effects, we conclude that the SMT is rather robust against deviations from equal SNP contributions.

Table 5 shows the performance of the SMT in the presence of 3-SNP recessive models, i.e., models in which the high-risk genotype is defined by homozygosity for the risk allele at each of three SNPs. The statistical interaction method of choice would be the analysis of either all two-way interactions or the analysis of three-way interaction. In the standard logistic regression setting, a 1-d.f. test for three-way interaction can be constructed by testing the allelic interaction parameter 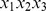. When dominance variance terms shall be considered, as well, eight different three-way interaction terms can be formulated and simultaneously tested. Pairwise interaction in each of three SNP pairs 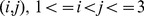 can be assessed either with a 1-d.f. allelic test for interaction (investigation of 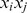) or a 4-d.f. genotypic test for interaction.

**Table 5:** Power values^a^ of SMT under 3-SNP-recessive models.

Model^a^	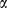 b	SMT^c^	Single-Marker^d^	2-SNP-1df^e^	2-SNP-4df^f^	3-SNP-1df^g^	3-SNP-8df^h^
REZ-A	5×10^−2^	1.00	1.00	1.00	0.99	0.71	0.72
	5×10^−4^	0.92	0.63	1.00	0.71	0.20	0.20
	5×10^−8^	0.86	0.08	0.54	0.07	0.00	0.00
REZ-B	5×10^−2^	0.69	0.47	1.00	0.86	0.85	0.80
	5×10^−4^	0.67	0.02	0.81	0.21	0.36	0.20
	5×10^−8^	0.62	0.00	0.10	0.00	0.00	0.00
REZ-C	5×10^−2^	0.88	0.85	0.95	0.88	0.79	0.84
	5×10^−4^	0.85	0.19	0.49	0.25	0.31	0.38
	5×10^−8^	0.80	0.01	0.01	0.01	0.00	0.02
REZ-D	5×10^−2^	1.00	0.99	0.86	0.86	0.53	0.43
	5×10^−4^	0.98	0.56	0.19	0.20	0.11	0.02
	5×10^−8^	0.43	0.02	0.01	0.02	0.00	0.00

a Different completely recessive 3-SNP models, with varying risk allele frequencies. REZ-A: 0.2,0.5,0.8; REZ-B: 0.2,0.3,0.4; REZ-C: 0.4,0.5,0.6; REZ-D: 0.6,0.7,0.8. Baseline penetrance was set to 0.03 and pentrances for 3-times recessive genotype were set to 0.2 (REZ-A), 0.7 (REZ-B), 0.1 (REZ-C), and 0.05 (REZ-D), respectively.

b Significance level.

c Power of supra-mulitplicativity test.

d Power of single-marker analysis, as computed from the most significant SNP, without correction for multiple testing.

e Power of 2-SNP logistic regression interaction test with 1 d.f. (allelic test), as obtained from the most significant SNP pair, without correction for multiple testing.

f Power of 2-SNP logistic regression interaction test with 4 d.f. (genotypic test), as obtained from the most significant SNP pair, without correction for multiple testing.

g Power of 3-SNP logistic regression interaction test with 1 d.f. (allelic test).

h Power of 3-SNP logistic regression interaction test with 8 d.f. (genotypic test).

At low significance levels, particularly at 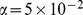, the 2-SNP 1-d.f. interaction tests outperforms the SMT, which is partially caused by the fact that power values for the pairwise tests are not corrected for the multiple testing of three pairs. At 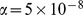, the SMT outperforms single-marker analysis and all standard interaction tests, under all four recessive models investigated. Under model REZ-C, for instance, power levels are below 0.02 for all standard tests while the SMT has remarkable power of 80%. This result might be surprising at first glance, but can be explained by the architecture of the SMT. The completely recessive model implies single-SNP dominance variation, two-way and three-way interactions. The interaction terms often will not reach significance, either because the effect is too small, but also because of the high number of degrees of freedom. The SMT, however, combines the evidence for two- and three-way interaction in a single test statistic that follows a 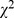-distribution with just one degree of freedom. In addition, the SMT takes into account that the effect directions of risk alleles are not altered, a fact that is not addressed when two-way and three-way interaction terms are combined in a standard regression test. Thus, the SMT can also be a powerful alternative when the goal is “just” interaction analysis of SNP sets of small size.

### Data Analysis

We applied the SMT to known Alzheimer’s disease (AD) susceptibility loci. According to the GWAS catalogue [1], there are currently 10 confirmed consensus AD genes [15]–[19]. These genes, together with the SNP showing strongest evidence for association, are APOE (rs429358), ABCA7 (rs3764650), CR1 (rs3818361), PICALM (rs3851179), CLU (rs11136000), BIN1 (rs744373), EPHA1 (rs11767557), CD2AP (rs9349407), CD33 (rs3865444) and MS4A (rs610932). We obtained genotypes for these SNPs from a previously unpublished late-onset Alzheimer’s disease (AD) Genome-wide case-control study, genotyped on the Illumina Omni1M micro-array. AD patients were recruited within the German Dementia Competence Network, DCN (http://www.kompetenznetz-demenzen.de) and at the interdisciplinary memory clinic of the Department of Psychiatry and Department of Neurology at the University Hospital in Bonn, Germany. Diagnosis of AD dementia was established according to NINCDA-ADRDA criteria [20]. All patients gave written informed consent for participation in the entire study protocol. After application of standard quality control procedures, 850,612 genotypes of 649 cases and 1,096 selected controls were available. We used the software IMPUTE2 [21] to impute into the February 2012 release of the 1,000 genomes project [22] and extracted the 10 AD SNPs for analysis.

As expected, our data shows overwhelming evidence for association of APOE with AD (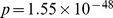, table 6). For the remaining SNPs, the level of significance that is reached with single-marker analysis is only moderate. However, for 8 out of 10 SNPs the risk allele is consistent with the risk allele reported in the GWAS catalogue, which suggests that the effects of the known SNPs are reflected in our data, but not significant because of a lack of power. Of note, for two SNPs, rs744373 (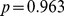) and rs3865444 (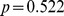), the effect direction is not in concordance with the GWAS catalogue. When the SMT is applied to a priori known SNPs, it is essential to count the number of risk alleles according to risk allele specification of the outside source and not according to the risk allele assignment that would follow from the data itself. Indeed, it cannot be expected that all susceptibility variants have the “correct” allele direction in a sample that is smaller than the samples from which the AD consensus SNPs were derived. Therefore, we computed the individual risk allele loads according to the GWAS consensus risk alleles.

**Table 6:** Single-marker analysis of GWAS Alzheimer’s disease susceptibility SNPs in independent data.

Chr	SNP	Position	Gene	Minor	Major	*p*-value	Odds Ratio
1	rs3818361	207784968	CR1	A	G	4.28×10^−4^	1.07
2	rs744373	127894615	BIN1	G	A	9.63×10^−1^	1.00
6	rs9349407	47453378	CD2AP	C	G	6.80×10^−2^	1.15
7	rs11767557	143109139	EPHA1	C	T	7.73×10^−2^	0.89
8	rs11136000	27464519	CLU	T	C	8.63×10^−2^	0.88
11	rs610932	59939307	MS4A	T	G	7.97×10^−1^	0.98
11	rs3851179	85868640	PICALM	T	C	3.85×10^−2^	0.86
19	rs3764650	1046520	ABCA7	G	T	3.81×10^−1^	1.11
19	rs429358	45411941	APOE	C	T	1.57×10^−48^	3.58
19	rs3865444	51727962	CD33	A	C	5.22×10^−1^	1.05

Prior to application of the SMT, we screened the data for SNP-dominance effects, and two- and three-way interactions. None of the dominance or pairwise interaction terms was nominally significant. Analysis of three-way interaction of 120 SNP triples was not significant after adjustment for multiple testing (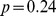).

**Figure 7:**
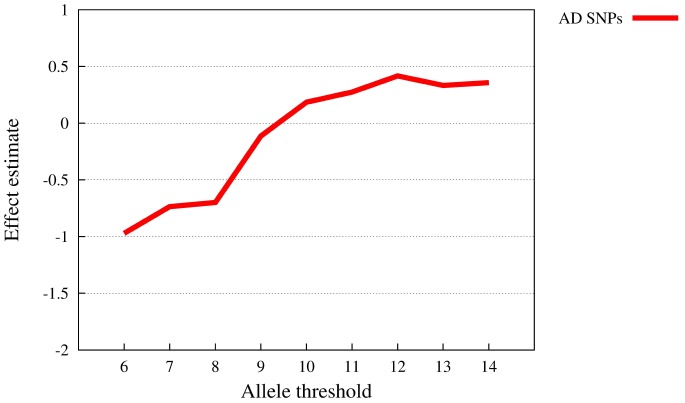
Curve of 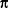-estimates for Alzheimer’s disease real data (10 susceptibility SNPs). The 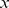-axis represents the allele threshold, the 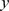-axis the supra-multiplicativity effect estimate.

Table 7 shows the results of the supra-mulitplicativity analysis. The allele thresholds 6,7 and 12 yield nominally significant p-values, 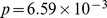, 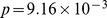 and 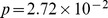, respectively. While the lower thresholds have effect estimates below zero (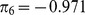, 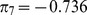), the higher threshold have an effect estimate above zero (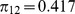). In other words, for thresholds 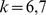 disease risk is lower than what would be expected under complete multiplicativity, while for 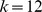 disease risk is “supra-multiplicative”. Thus, the effect directions are consistent with the notion of a threshold model, disease risk increases over-proportionally with higher risk allele loads. However, a sharply defined threshold, as observable with simulated data (figure 5), is not detectable. Instead, the effect estimates 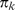 grow gradually with increasing threshold 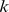 (figure 7). For illustrative purposes, table 6 also contains odds ratios computed from a two-by-two contingency table with case-control status and the number of individuals with a risk allele load below/above the threshold as attributes. These odds ratios also grow gradually with increasing allele threshold.

**Table 7:** Application of SMT to Alzheimer’s disease susceptibility SNPs.

Alleleload^a^	*p*-value^b^	*π* c	*se* d	OR^e^	seOR^f^	Freq_Cases^g^	Freq_Control^h^
6	2.23×10^−2^	−0.971	0.370	0.852	0.737	0.981	0.983
7	6059×10^−3^	−0.736	0.239	1.205	0.433	0.948	0.938
8	9.16×10^−4^	−0.700	0.189	1.270	0.294	0.879	0.851
9	5348×10^−1^	−0.114	0.171	1.636	0.232	0.787	0.693
10	3.20×10^−1^	0.185	0.167	1.818	0.203	0.643	0.498
11	1.34×10^−a^	0.274	0.165	2.046	0.205	0.457	0.292
12	2.72×10^−2^	0.417	0.170	2.315	0.244	0.279	0.144
13	1.51×10^−1^	0.333	0.209	2.549	0.353	0.128	0.054
14	3.09×10^−^	0.357	0.318	3.025	0.593	0.048	0.017

a Risk allele threshold 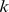 under investigation.

b Uncorrected p-value for given threshold.

c Effect estimate 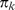 for indicator variable 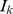 at threshold 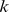.

d Corresponding standard error.

e Odds ratio as computed from two-by-two case-control table with number of cases/control with a risk allele load above and below the threshold.

f Corresponding standard error.

g Frequency of cases above the allele load threshold.

h Frequency of controls above the allele load threshold.

In summary, we see an over-proportional increase in disease risk, along with growing risk allele load. Since the supra-multiplicativity is not attributable to lower order interactions terms, we can state that the deviation from multiplicativity would have gone undetected without the SMT.

## Discussion

We presented the SMT as a test for deviation from multiplicativity. It can be applied to sets of a size of up to 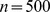 SNPs and allows joint investigation of all 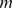-way interactions, 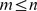, by a single parameter. With 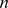 SNPs, there are 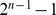 allelic interaction terms 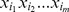 that theoretically could be analyzed in a regression framework. With growing 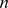, such systematic investigation quickly reaches the limit of computability. The SMT, however, can simultaneously assess interactions of different orders. In addition, the formulation as a one parameter test improves power via the reduction of degrees of freedom and by reduction of the multiple testing burden. The test is powerful under two assumptions: First, effect *sizes* of SNP alleles have to depend on the combination of genotypes at other disease SNPs, but, effect *directions* must not depend on the genotypes at other model SNPs. Indeed, the indicator parameter counts the number of risk alleles of an individual. As a consequence, the SMT will lose power when effect directions can be reverted. Second, a substantial part of the SNPs under investigation must deviate from multiplicativity. This assumption might be considered to be a strong one, but is, on the other hand, fulfilled by biologically plausible models which at the same time are easily specified. In particular, limiting pathway liability models (LPLMs) lead to strong supra-multiplicativity which can be detected by the SMT, as shown in our simulation study. Moreover, the SMT remained powerful when we altered the LPLMs such that marginal SNP effects were allowed to vary substantially. In addition, we exemplified the usefulness of the SMT for small SNP sets by showing that it is much more powerful than standard interaction tests under 3-SNP-recessive models.

Zuk et al. [6] have proposed LPLMs as a possible explanation for missing evidence for epistasis in GWAS studies. With the SMT it is now possible to systematically screen GWAS susceptibility SNPs for their involvement in a LPLM. More generally, our approach can be used as a tool to screen pre-defined SNP sets for supra-multiplicativity. Thereby, it has the potential to assess the presence of simultaneous effects of known SNPs and to contribute to the judgement of their joint relevance. In this context, previously confirmed, particular 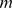-way interactions can be used as model covariates, in order to detect supra-multiplicativity on top of such interactions.

Our analysis of known Alzheimer’s disease (AD) susceptibility SNPs in previously unpublished data revealed supra-multiplicativity (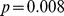) that was neither attributable to SNP dominance, nor to pairwise or three-way interaction effects. A sharp risk allele threshold as it would be characteristic of LPLMs was not identifiable. Instead, deviation from multiplicativity developed gradually with increasing allele load, and, therefore, was not detectable with standard interaction analysis.

Confirmed supra-multiplicativity of risk factors is of potential relevance in various fields. In the presence of supra-multiplicativity, prediction of disease risk can be improved when the joint distribution of risk factors is fully modeled in contrast to prediction based on multiplication of risk factors. For application in clinical practice, it is important that all risk factors as such, as well as their amount of supra-multiplicativity, are confirmed by independent studies. In addition, independent studies would have to specifically investigate whether prediction can be improved over prediction based on the domineering APOE locus.

Supra-multiplicativity is perhaps even more important for treatment invention and drug development than it is for risk prediction. Supra-multiplicative risk factors imply over-proportionally strong disease risk when they occur together. Conversely, this means that disease risk can be substantially reduced when the effects on phenotype of only a portion of the risk factors can be blocked. Under a risk threshold model, for instance, disease risk can be reduced to a base-line level when the number of still effective risk factors can be reduced so far that the number of still active risk factors is below the risk threshold. In particular, this means that for a complex disease it might not be necessary to have an antidote for all risk factors, it might be sufficient to inactivate just some of these. Stringent statistical evidence for strong supra-multiplicativity of the risk factors of the disease under investigation, however, is an essential prerequisite for such a perspective.

## Methods

Our work got its stimulus from the manuscript of Zuk et al. [6] who suggested limiting pathway liability models (LPLMs) as an explanation for the missing evidence for interaction in GWAS studies. An LPLM can be defined as follows: Consider a set of 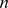 SNPs, and specify for each SNP its risk allele. Let 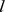, 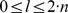 be the number of risk alleles of an individual. Let 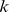, 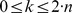 be a liability threshold. For individuals with 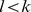, the risk to be a case is set to be equal to a baseline penetrance 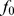, while for individuals with 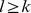 risk alleles, an increased penetrance 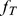 applies. It is typically assumed that 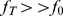 and that 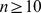. By design, such a model leads to marginal SNP effects and also to 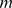-way interaction effects for all 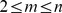. Indeed, the effect of the risk allele of SNP 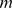, 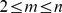, depends on the number of risk alleles present at SNPs 1 to 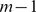. Since the interaction effects are distributed over all orders, particular single interaction terms are rather small. As a consequence, search for, for instance, pairwise interaction is not the method of choice in the presence of a LPLM. Motivated by this, we construct a one degree of freedom (d.f.) test for deviation from multiplicativity for a set of 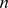 SNPs that simultaneously addresses all 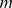-way interaction effects, 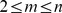. An implementation can be found in our software package INTERSNP [23] (http://intersnp.meb.uni-bonn.de).

We consider a set of 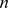 SNPs with corresponding parameters 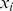, 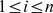, which follow the allele coding used in the logistic regression framework described elsewhere [24]. We introduce a series of indicator parameters 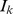, 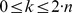, where 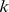 is a liability threshold that shall be investigated. In case an individual has 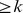 risk alleles, we set 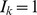, otherwise we set 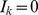. Next, we test the liability threshold 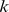 by comparing
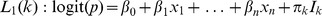
against



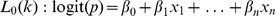



In other words, we use the marginal effects of the SNPs as covariates and investigate if the disease risk increases sharply when 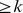 risk alleles are present. Since the optimal cut-off is not known in advance, all values of 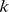 have to be tested. We compute the final p-value 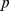 as 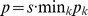 where 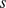 is the number of cut-offs 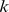 for which there are individuals both above and below the threshold. The method will be conservative since the tests are not independent for different values of 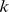. In the results section, we will show that the Bonferroni-correction is, nevertheless, more than acceptable for practical purposes. We note that the suggested test allows the inclusion of further covariate parameters. Adaption to quantitative traits is also straightforward.

By design, the proposed test can detects various types of deviation from multiplicativity. Therefore, we call it a supra-multiplicativity test (SMT). The SMT is constructed to optimize power in the presence of LPLMs. More generally, one can expect the SMT to be powerful when a substantial portion of the sub sets of the SNP set under investigation deviate from multiplicativity. In addition, effect directions should not be reverted in combination with other risk alleles since the test is build up on risk allele counts per individual.

### Simulation Set-up

#### Null models

Let 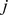 be the number of risk alleles, 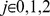, at a given SNP and let 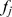 be the respective penetrance. To investigate the proposed test under the null hypothesis “no deviation from multiplicativity”, we simulated case-control data for 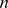, 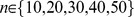 disease SNPs under a completely multiplicative model. We considered 1,000 permutation replicates for each choice of *n*, and, for each replicate, randomly selected allele frequencies from a uniform distribution and randomly assigned relative risk values 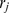 from 1.2 to 1.5. In order to investigate to SMT in the presence of covariates, we considered “semi-null” models, i.e., models with deviation from multiplicativity that was attributable either to single SNP dominance effects or to two-way interaction. We simulated 30 SNPs. In model 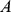, 15 SNPs were simulated under a model of recessive type, the relative risk for homozygote carriers was set to 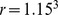. In model 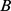, 7 SNP pairs were simulated under a double-recessive model, i.e., the relative risk was set to be 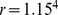 for the two-locus genotype with 4 risk alleles. All other effects were combined multiplicatively.

#### LPLMs

In order to assess power of the SMT, we investigated limiting pathway liability models (LPLMs) as suggested by Zuk et al. [6]. Let 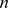 be the number of model SNPs and let 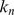 be a respective allele load threshold. In our set-up, 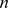 ranged from 10 to 50 in step sizes of 10 and the threshold 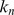 was chosen such that about 1.5% of the general population had an allele load equal to or above the threshold 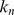. For individuals below 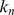, a baseline penetrance value of 0.03 was assumed. For individuals above the threshold, we assumed a strongly increased penetrance 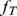, where 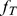 was chosen from 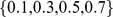. For each parameter constellation 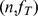, we simulated 1,000 data sets with 3,000 cases and 3,000 controls and estimated empirical power at the level 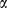 as the portion of simulated data sets significant at 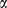. We decided to present power at the level 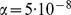, since an exhaustive search over all subsets of a given set of SNPs quickly leads to a high number of tests. In addition, analysis of 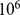 is a number of tests that can be analyzed within a reasonable time frame with our implementation. In practice, a less stringent 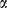 might be considered sufficient, depending on the number of models that are actually tested, cf. also the section “Data analysis”. Power of the liability was not only analyzed for the entire SNP set, but also for marker subsets of all possible sizes, in order to mimic the situation that not all SNPs belonging to a threshold model will be available or known. For a set of 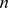 SNPs, the number of subsets of size 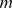 can be enormously high. Therefore, we could not analyze all subsets, but investigated the “first” 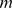 SNPs, in the arbitrary order implied by the set-up, to assess power for subsets of size 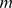. This procedure has also the advantage of improved comparability when moving from subset size 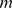 to subset size 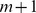.

We also investigated the potential impact of incomplete SNP coverage on the power of the SMT. To this purpose, we assumed that the true causal SNPs of the 30-SNP LPLM (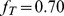) are not available, but only tagged by proxy SNPs with an 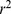 of 0.80. After genotype imputation, such approximation of causal SNPs by SNPs in linkage disequilibrium is realistic.

#### Modified threshold models

The LPLMs suggested in [6] might be considered to be to simplistic. In particular, differences in the contribution of individual SNPs should be allowed in multi-SNP models [9]. Therefore, we modified the 30-SNP LPLM (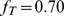) from above as follows. We maintained the load threshold from before, but varied the weighting of the risk allele contributions from different SNPs. For one third of the SNPs, the risk allele counts were weighted with a factor of 0.5, for another third of the SNPs, the risk allele counts were weighted with a factor of 1, and the remaining SNPs were weighted with a factor of 2.

#### Recessive models

We investigated “completely recessive” models defined by three SNPs. A baseline penetrance of 0.03 was assumed. Only individuals which were homozygous for the risk allele at all three SNPs were assigned an increased penetrance. We simulated data under different scenarios, defined by varying population allele frequencies and risk genotype penetrance values. In detail, we simulated the model REZ-A with allele frequencies of the three SNPs of 0.2, 0.5 and 0.8, respectively, and a penetrance of 0.20 for the high risk 3-SNP-genotype. Model REZ-B was defined by allele frequencies 0.2, 0.3 and 0.4 and a high risk genotype penetrance of 0.70, model REZ-C was defined by allele frequencies 0.4, 0.5 and 0.6 and a high risk genotype penetrance of 0.10, and, model REZ-D was defined by allele frequencies 0.6, 0.7 and 0.8 and a high risk genotype penetrance of 0.05. The resulting allelic relative risks ranged from 1.05 to 1.19 under model REZ-A, from 1.03 to 1.06 under REZ-B, from 1.05 to 1.08 under REZ-C, and, from 1.10 to 1.13 under REZ-D.
