# Correction: A One-Degree-of-Freedom Test for Supra-Multiplicativity of SNP Effects

**DOI:** 10.1371/journal.pone.0114890

**Published:** 2014-11-26

**Authors:** The PLOS ONE Staff

In Table 7, several values in the *p*-value^b^ column are incorrect due to errors that occurred during the typesetting process. Please see the corrected Table 7 here.

**Table 7:** Application of SMT to Alzheimer’s disease susceptibility SNPs.

Allele load^a^	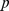 -value^b^	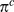	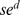	OR^e^	seOR^f^	Freq_Cases^g^	Freq_Control^h^
6	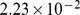	-0.971	0.370	0.852	0.737	0.981	0.983
7	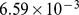	-0.736	0.239	1.205	0.433	0.948	0.938
8	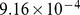	-0.700	0.189	1.270	0.294	0.879	0.851
9	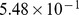	-0.114	0.171	1.636	0.232	0.787	0.693
10	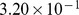	0.185	0.167	1.818	0.203	0.643	0.498
11	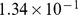	0.274	0.165	2.046	0.205	0.457	0.292
12	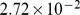	0.417	0.170	2.315	0.244	0.279	0.144
13	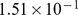	0.333	0.209	2.549	0.353	0.128	0.054
14	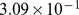	0.357	0.318	3.025	0.593	0.048	0.017
